# OnabotulinumtoxinA in the treatment of refractory chronic cluster headache

**DOI:** 10.1186/s10194-018-0874-y

**Published:** 2018-06-19

**Authors:** Christian Lampl, Mirjam Rudolph, Elisabeth Bräutigam

**Affiliations:** 1 Headache Medical Center, Ordensklinikum Linz Barmherzige Schwestern, Linz, Austria; 2 Headache Medical Center, Department of Radio-Oncology, Ordensklinikum Linz Barmherzige Schwestern, Linz, Austria

**Keywords:** Headache, Cluster headache, Refractoriness, Onabotulinumtoxin a, Prophylactic treatment

## Abstract

**Background:**

Cluster headache (CH) is a clinically well-defined primary headache disorder, approximately 20% of cluster headache sufferers experience recurrent attacks without periods of significant remission. For the treatment of chronic cluster headache (CCH) only limited therapeutic options are available.

**Methods:**

A potential refractory CCH patient group was identified according to the clinical definition of rCCH based on the consensus statement of the European Headache Federation (EHF). Treatment with OnabotulinumtoxinA (BoNT-A; Botox®, 150 Allergan IU) was done according to the PREEMPT study protocol. A standardized headache diary was used for recording frequency, duration of attacks and pain intensity. To assess personal burden the HIT-6 and the Hospital Anxiety and Depression scale was used. Primary outcome measure was a > 50% reduction in headache minutes.

**Results:**

Seventeen male patients suffering from rCCH, aged 32 ± 11 (mean ± SD) years, presenting a mean disease duration of 6.6 years completed the study of 28 weeks. The cut-off point of > 50% reduction in headache minutes as positive result was reached in 58.8%, 29.4% experienced an improvement of 30–50%. Mean frequency of headache days dropped from 28.2 to 11.8 days at week 24 (*p* = 0.0001; 95% CI -21.33 to − 11.61;). Intensity of remaining attacks was also reduced significantly. Headache disability scores showed a trend to improvement after BoNT-A.

**Conclusions:**

Encouraging results for the treatment with BoNT-A in rCCH patients were observed in our study population.

## Background

Cluster headache (CH) is a clinically well-defined primary headache disorder occurring in both episodic and chronic forms. Chronic cluster headache (CCH) is a rare condition - approximately 20% of cluster headache sufferers experience recurrent attacks without periods of significant remission [1]. CCH may be unremitting from onset or evolve from episodic CH [2]. The evolution to CCH has been reported to occur in 3.8% to 12.9% of episodic CH sufferers [3]. According to the IHS classification ICHD-3rd version [4] CCH fulfils criteria for episodic cluster headache with attacks recurring without a remission period or with remissions lasting < 3 month for at least one year. For the treatment of CCH only limited therapeutic options are available so far. CCH sufferers often overuse symptomatic medications to treat CH attacks and may develop medication overuse headache (MOH) in addition. To avoid this risk it is worth noting that the preventive treatment of CCH is essential. The decision about how to treat is largely based on clinical experience. For those with CCH preventive treatments are used for an indefinite period of time at least until the patient has been in remission without attacks for 6 months. It is often used in conjunction with a transitional agent and in combinations. Preventive medication should be preferably used as monotherapy but combinations of suggested preventive treatments are recommended especially if one preventive treatment decreases the attack frequency but does not control the situation satisfactorily, upon the physician’ s decision [5].

Botulinum Neurotoxin Type A (OnabotulinumtoxinA; BoNT-A) is well established in the treatment of chronic migraine (for review [6]). The intention of this study was to evaluate the efficacy and tolerability of BoNT-A as add-on therapy in refractory CCH (rCCH) patients.

## Methods

**Fig. 1:**
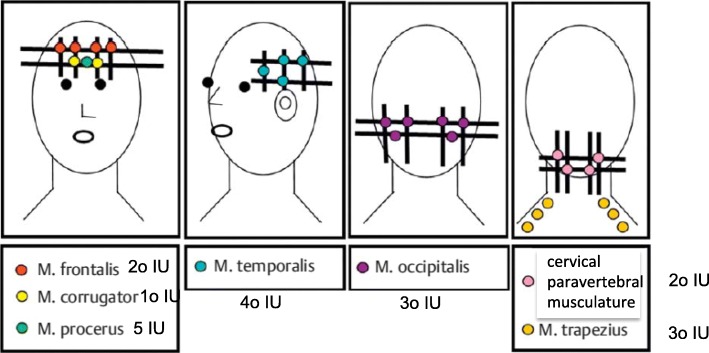
Injection procedure of OnabotulinumtoxinA according to the PREEMPT study protocol

Potential rCCH patients were identified from the database of our headache medical centre and from the registry of Linde Austria. The clinical definition of rCCH was based on the consensus statement of the European Headache Federation (EHF) [5]. This study was performed as an open label, non-randomised, single-centre study. Patients between 18 and 60 years of age were included. Exclusion criteria were those for BoNT-A therapy (e.g., generalised muscle weakness, myasthenia gravis, gravidity or known antibodies against botulinum toxin), symptomatic CCH and patients with occipital nerve stimulation (ONS) in situ. After a detailed elucidation of the procedure, injection sites, possible side effects and communication about the off label use of BoNT-A eligible patients undersigned informed consent. BoNT-A (Botox®, 150 Allergan IU) was used according to the Phase 3 REsearch Evaluating Migraine Prophylaxis Therapy (PREEMPT) [7, 8] study protocol (Fig. 1). A standardized headache diary was used including frequency (days/month), duration of attacks (min/attacks/day) and pain intensity (numeric rating scale, NRS). Pain duration was measured with a stop-watch (either commercial one or with mobile phone stop-watch). To assess personal burden patients were asked to fill out the six-item Headache Impact Test (HIT-6) and the Hospital Anxiety and Depression scale (HADS). The HIT 6 was designed to provide a global measure of adverse headache impact [9, 10] and was developed for use in screening and monitoring patients with headaches in both clinical practice and clinical research. The HADS [11] consists of two subscales: HADS-Anxiety (HADS-A) and HADS-Depression (HADS-D) - each of seven items. In response to each item, participants report their subjective experience at the end of week 4 (baseline), week 12 and week 24, rating it 0–3 (3 indicating maximum symptom severity). The sum of each subscale has a potential range of 0–21. As recommended in the original description [11] we took a threshold of 11 on the respective subscale to indicate caseness for anxiety or depression.

Observational period was 28 weeks, 4 weeks pre-treatment (baseline) and 24 weeks treatment phase. Headache frequency and duration was recorded per day and added at the end of each 4 weeks period (baseline; mean of each 4 weeks period at week 12 and mean of each 4 weeks period at week 24 of the treatment phase). 150 Allergan IU was injected in week 1 after baseline and in week 12. BoNT-A was given as add-on therapy, prior acute and prophylactic treatment was allowed to continue. Any change in medication was recorded. Mean and median values of baseline and week 24 were compared using Wilcoxon Signed Ranks tests and a statistically significant result set at the 95% level (*p* = 0.05). Statistical Package for Social Studies (SPSS) version 17.0 (Aug 23, 2008) (SPSS Inc., Chicago, IL) was used to analyse our data. The study got approval by the local ethic committee (EK 4217; EudraCT No: 2017–003873-33). Primary endpoints were the change in headache days, change in headache minutes comparing baseline and week 24, with a cut-off point of 50% or greater improvement. Secondary outcomes were achieving a 30% to 50% improvement in headache minutes, change in pain intensity of the attacks, change in HIT6 and HADS scores and change in daily analgesics and prophylactic treatment.

## Results

In total 27 patients (21 from the Linde data base, 6 from our headache center; f:m/5:16) were identified. Twentyone patients were contacted, all of them agreed to participate. Due to the exclusion criteria 19 male patients with rCCH, aged 32 ± 11 (mean ± SD) years, with a mean disease duration of 6.6 years were enrolled in the study and received initial treatment with BoNT-A. Two patients had to be excluded because of missing data according to the study protocol. Table 1 shows which treatment and which dose was tested before the patients were classified as rCCH. The results of BoNT-A treatment are summarized in Table 2. The cut-off point of > 50% as positive result was reached in 10/17 patients (58.8%). Three patients experienced total cessation of attacks within the study period; five patients (29.4%) achieved a 30–50% reduction of attack minutes. In 2 patients no improvement was observed. Mean frequency of headache days dropped significantly to − 16 days (*p* = 0.0001; 95%CI -21.33 to − 11.61;). Mean headache minutes decreased to − 1.329 (p = 0.0001; 95% CI -1.778.82 to − 878.24). Intensity of remaining attacks was also reduced significantly. The mean primary pain score at subjects’ referral was 7.80 ± 1.25. It dropped to a mean of 3.85 ± 1.11 (*p* = 0.0038; 95% CI -6.85 to − 0.1). Percentage of pain score reduction did not correlate with the subjects’ duration of illness (correlation coefficient = 0.113, *p* = 0.756). Headache disability scores showed a trend to improvement after BoNT-A (Table 3), HIT-6 showed a mean change of − 12.7 points (*p* = 0.021; 95% CI -21,3 to − 1.8), HADS-A a mean change of − 2.2 (*p* = 0.078; 95% CI -6.1 to − 0.1); HADS-D a mean change of − 3.2 (*p* = 0.063; 95% CI -8.2 to − 0.1). Adverse events were reported in 7 patients: Four observed an eyebrow ptosis, 3 patients mentioned a transient worsening in headache before improvement. All adverse events were rated as mild by patients and transient in nature. Preventive medication could be stopped in 6 patients (4 with topiramate, 2 with verapamil). In 4 patients dosage of verapamil could be bisected, 2 patients had no prophylactic treatment at baseline and 5 patients recorded no change in their prophylactic medication.

**Table 1:** Demographic details, medication used to manage CCH before enrolment in the study

patients ID	triptans	oxygen	mid analgesics	Verapamil (mg)		Lithium (mg)*		Propranolol (mg)*		Amitriptyline (mg)*		Topiramate (mg)*		Corticosteroids (mg)**
				hd	ld	hd	ld	hd	ld	hd	ld	hd	ld	
1	✓	12 l	✓	480	480	X	X	120	80	X	X	200	200	25
2	✓	15 l	✓	480	480	450	X	120	80	?	10	100	100	X
3	✓	15 l	✓	600	480	450	X	120	X	X	X	200	200	X
4	✓	15 l	✓	600	240	X	X	120	X	75	75	100	X	X
5	✓	12 l	✓	720	480	X	X	80	40	X	X	100	X	75
6	✓	12 l	✓	240	240	900	450	240	X	25	25	X	X	10
7	✓	12 l	✓	840	480	X	240	X	X	10	X	100	100	50
8	✓	10 l	✓	480	480	X	X	40	X	X	X	100	100	X
9	✓	12 l	✓	600	X	450	X	80	X	?	X	100	X	50
10	✓	15 l	✓	240	240	450	X	120	X	25	X	X	X	50
11	✓	15 l	✓	480	240	450	450	X	X	X	X	100	100	X
12	✓	15 l	✓	480	480	X	X	120	X	X	X	100	100	50
13	✓	15 l	✓	600	600	X	X	40	X	X	X	200	200	25
14	✓	15 l	✓	600	X	450	X	X	X	?	X	150	X	X
15	✓	12 l	✓	600	480	450	X	X	X	X	X	200	200	X
16	✓	15 l	✓	240	240	450	450	X	X	X	X	100	100	25
17	✓	10 l	✓	720	X	450	X	?	X	X	X	200	200	X

l = litre; hd = highest dosage used; ld = last dosage used before enrolled in the study; mg = milligram; ✓□ = used; X = not used;? = not known; ** no longer than 1 month

**Table 2:** Demographic details, headache scores pre- and post- treatment with OnabotulinumtoxinA

patients ID	duration/y	baseline		treatment phase					patient subjective estimate of response %^b^
		frequency d/mo	duration min	week 12		week 24		improvement %^b^	
				frequency d/mo^a^	sum of min/ bout^a^	frequency d/mo^a^	sum of min bout^a^		
1	7	30	2.250	17	1.755	14	1.320	41.3	50
2	4	25	1.632	8	337	0	0	100	90–100
3	3	28	1.328	11	472	7	221	83.3	60–70
4	5	29	2.598	0	0	0	0	100	100
5	5	30	3.148	12	1.065	8	531	83.1	75
6	5	30	1.912	8	867	5	312	83,6	70
7	9	28	2.491	12	1.289	7	1.333	46.4	50
8	7	30	1.870	8	473	6	328	82.4	70–80
9	7	25	1.140	13	833	11	618	45.8	50
10	4	27	3.456	14	2.855	12	1.565	54.7	50
11	3	30	1.080	30	1.418	30	1.377	0	0
12	3	30	3.020	16	1.844	14	813	73	50
13	3	30	2.280	29	2.065	30	2.555	0	0
14	6	28	1.680	0	0	0	0	100	100
15	4	27	2.479	18	1.349	22	1.662	32,9	20–30
16	2	30	3.040	17	1.364	14	839	72.4	50–70
17	5	23	1.855	21	989	20	1.200	35,3	20
Mean	5	28.2	2.192	13.8	1.117	11.8	863	62	
median	5	29	2.250	13.5	1.065	11	813	62	
(range)	(2–9)	(23–30)	(1.080–3.148)	(0–30)	(0–2.855)	(0–30)	(0–2.555)	(0–100)	

*pre* = before treatment (=baseline), *y* = year; *mo* = month, *w* = week, *d* = day, *min* = minutes; ^a^ = mean of every 4 weeks over past 12 weeks;

^b^= baseline vs week 24

**Table 3:** Headache-associated disability scores pre- and post- treatment with OnabotulinumtoxinA

	HIT- 6 (36–76)			HADS - A (0–21)			HADS - D (0–21)		
patients ID	pre	w 24	change in score	pre	w 24	change in score	pre	w 24	change in score
1	70	50	20	18	15	3	8	6	2
2	58	36	22	15	9	6	12	8	4
3	67	47	20	17	15	2	15	12	3
4	76	42	34	15	6	9	6	3	3
5	78	58	20	15	12	3	9	4	5
6	58	52	6	11	8	3	10	3	7
7	63	58	5	13	11	2	10	7	3
8	63	42	21	13	12	1	8	2	6
9	57	42	13	12	11	1	12	11	1
10	78	68	10	11	12	-1	11	9	2
11	65	68	−3	13	15	−2	14	15	−1
12	68	57	11	11	12	−1	11	5	6
13	65	65	0	18	21	−3	16	16	0
14	56	36	20	12	6	6	4	0	4
15	53	57	−4	14	12	2	15	12	3
16	68	54	14	14	9	5	12	9	3
17	76	72	4	12	11	1	14	11	3
Mean (95% CI)	65.8	53.1	−12.7 (−21.3; −1.8)	13.8	11.6	−2.2 (−6.1; −0.1)	11.0	7.8	−3.2 (−8.2; −0.1)
median (range)	65 (53–78)	54 (36–72)	13 (− 3–34)	13 (11–18)	12 (6–21)	2 (− 2–9)	11 (6–16)	8 (0–16)	3 (− 1–7)

*pre* = before treatment (=baseline), *w* = week; *HIT-6* = Headache Impact Test, *HADS*=Hospital Anxiety and Depression Scale, *A* = anxiety, *D* = depression

## Discussion

BoNT-A was highly effective in rCCH in 10/17 patients. Three “super-responders” were identified as well as a median reduction in headache minutes of 62% in all patients. Nine patients (52.9%) reported a > 70% improvement in headache minutes. Clinically significant improvements were also seen in both HIT-6 and HADS scores. Our patients tried at least 3 preventive medications in recommended dosage and had suffered from CCH for a mean of 6.6 years at the time of BoNT-A treatment. This refractory nature of the group means that it is doubtful that our observations are due to spontaneous remission.

The efficacy of BoNT-A as CCH prophylaxis has so far been studied only in single case reports [12–14] and in one open trial with a group of 12 CH patients following a standardised injection scheme [15], but not the PREEMPT study protocol. Using 50 IU of BoNT-A the German study group observed a reduction of attack frequency in 25% (3/12) of all study patients and in 33% (3/9) of patients with CCH. Interestingly they observed a better approach in the majority of patients who suffered from CCH for a shorter period (1.5–2 years) than patients with a longer duration of CCH (3–12 years). That could not be observed in our study population. Although the aetiology and pathophysiology of CH is still not completely understood we do have evidence from PET studies [16, 17], voxel-based morphometry [18] and stereotactic hypothalamic deep brain stimulation [19, 20], that a dysfunction of the ipsilateral posterior hypothalamus may cause a secondary activation of the trigemino-autonomic brainstem pathways [21]. This activation leads to the release of calcitonin gene-related peptide (CGRP) a potent vasodilator and neurotransmitter [22]. The activation of the trigeminal system during CH attack is indicated by the elevation of CGRP plasma levels in the external jugular vein [23]. CGRP plasma levels are also elevated interictally in episodic CH patients in the bout compared to outside the bout [23]. The question now is how BoNT-A interferes with the pathophysiology of CCH and whether it is responsible for the positive effects observed in our study. BoNT-A inhibits the release of CGRP from peripheral trigeminal neurons and consequently reduces the CGRP-mediated trigeminal sensitization in migraine [24, 25]. Furthermore, it was suggested that BoNT-A exhibits its actions in pain and migraine by reaching dural trigeminal afferents [26, 27]. Due to the ability of BoNT-A to undergo retrograde axonal transport to the CNS [28] neurotransmitters like Substance P [29] or CGRP [30] might be modulated not only locally at the injection site but also at anatomically connected sites in the trigeminal terminals.

The effects of BoNT-A in the cranial dura could be reconstructed as follows [31]: after peripheral injection BoNT-A is taken up by sensory nerve endings and axonally transported to trigeminal ganglion. After transcytosis the toxin reaches dural nerve endings containing CGRP, suppresses the CGRP-mediated sensitization of the trigeminovascular system, resulting in reduction in local neurogenic inflammation. At present this seems to be the most convincing hypothesis of the action of BoNT-A in migraine and CH. Moreover modulation of neurotransmitter release at higher order neurons could contribute to pain attenuation as brain Gamma-aminobutyric acid (GABA) concentration may be correlated to migraine intensity [32]. This could also be a mechanism in cluster headache patients to modulate pain intensity.

Although we observed encouraging results for the treatment with BoNT-A in rCCH patients, major limitations of this study arise from its open design and the small patient number. Previous studies of BoNT-A have also reported a significant placebo response; therefore we cannot exclude this as a potential confounding factor in our outcomes as well as effects of concomitant preventive therapy. However, the good response rates and consistent efficacy of repeated BoNT-A application in our study population with an extraordinary pain condition suggest that the response to BoNT-A in this series entirely cannot be attributed to a placebo response. Further multicentre studies with higher patient numbers will be needed to further clarify if BoNT-A is effective in the prophylactic treatment of rCCH. As the syndrome is quite rare, it is still essential to collect and publish large case series regarding clinical manifestations and treatment options.

## Conclusions

Our data suggest that the injection of BoNT-A could be beneficial as add-on therapy in patients with otherwise rCCH. However, these preliminary results has to be confirmed in double-blind, randomised, controlled studies. Especially patient characteristics that predict benefit from BoNT-A treatment should be identified. In our experience it is essential to use the PREEMPT study protocol for the injection procedure.

## Abbreviations

BoNT-A: OnabotulinumtoxinA
CCH: Chronic Cluster Headache
CGRP: Calcitonin gene-related peptide
CH: Cluster Headache
GABA: Gamma-aminobutyric acid
HADS: Hospital Anxiety and Depression scale
HIT-6: Headache Impact Test
ICHD: International Classification of Headache Disorders
IU: International Units
MOH: Medication Overuse Headache
NRS: Numeric Rating Scale
PREEMPT: Phase 3 REsearch Evaluating Migraine Prophylaxis Therapy –
rCCH: Refractory Chronic Cluster Headache
SPSS: Statistical Package for Social Studies
